# Effects of fenclorim on rice physiology, gene transcription and pretilachlor detoxification ability

**DOI:** 10.1186/s12870-020-2304-y

**Published:** 2020-03-06

**Authors:** Lifeng Hu, Ying Yao, Ruwen Cai, Lang Pan, Kailin Liu, Lianyang Bai

**Affiliations:** 1 College of Plant Protection, Hunan Agricultural University, Changsha, 410128 People’s Republic of China; 2 Collaborative Innovation Center of Farmland Weeds Control, Hunan University of Humanities, Loudi, 417000 People’s Republic of China; 3 Hunan Agricultural Biotechnology Research Institute, Hunan Academy of Agricultural Sciences, Changsha, 410125 People’s Republic of China

**Keywords:** Safener, Rice, Antioxidation, Metabolism, Transcriptomics

## Abstract

**Background:**

Fenclorim (Fen) can effectively protect rice from pretilachlor (Pre) injury, but its effects on rice have not been formally evaluated; thus, the Fen mode of action for alleviating the phytotoxicity caused by Pre in rice is not clear. This study aimed to examine the biochemical and physiological effects of Fen on rice and to determine the changes induced by Fen at the transcriptome level.

**Result:**

The chlorophyll content of rice plants was significantly affected by Pre but not by Fen. The activity of oxidative stress enzymes showed that Fen did not elicit any changes in oxidative stress; however, it reduced lipid peroxidation and oxidative damage induced by Pre. Fen did not affect the uptake of Pre but did affect its persistence in rice. In a transcriptome experiment, Fen upregulated genes in a detoxification pathway. Overall, 25 genes related to detoxification were identified, including P450, GST, and GT. Moreover, qRT-PCR analysis showed that four P450 genes, *CYP71Y83*, *CYP71K14*, *CYP734A2* and *CYP71D55*, and two GST genes, *GSTU16* and *GSTF5*, were upregulated by Fen and/or Pre.

**Conclusion:**

Our work indicates that Fen acts in antioxidative defense in addition to enhancing the metabolism of herbicides in rice.

## Background

It is well known that herbicides, which constitute the primary method of weed control in modern agriculture practices, have played a crucial role in controlling farmland weeds and subsequently reducing labor intensity. While herbicides can help increase crop yields, they can also pose risks to crops that are sensitive to them; for this reason, safeners were developed to increase crop selectivity [1]. Herbicides are biologically active xenobiotic compounds, and their overuse may disrupt the normal biochemical and physiological processes of plants. Each herbicide has at least one specific target site, and the inhibition of the target site activity is the main mode of action of many herbicides. In addition, many studies have shown that reactive oxygen species (ROS) can accumulate in plant cells as a secondary effect when the cells are exposed to herbicides [2, 3]. Excess ROS can damage plant cells by leading to membrane lipid peroxidation, protein oxidation, inhibition of enzyme activity, DNA and RNA damage, etc. and can even result in cell death [3–8].

Over the course of long-term evolution, plants have evolved the ability to minimize herbicide toxicity. One of their protective mechanisms is the antioxidant system, which is composed of a variety of enzymes, including superoxide dismutase (SOD), peroxidase (POD), catalase (CAT), ascorbate peroxidase (APX) and other enzymes, for protecting against oxidative damage [7]. Antioxidant enzyme activity has been proven to be related to herbicide tolerance in a variety of plants [5, 6, 8, 9].

Another mechanism is a detoxification system also known as the xenome, which removes complex exogenous compounds [10]. This detoxification system is composed of large families of enzymes, such as the P450s, GTs, GSTs, ABC transporters and other enzymes or transporters, and the process by which it works can be divided into four phases: phase I, hydrolysis or oxidation; phase II, conjugation; phase III, sequestration; and phase IV, catabolism [1, 11]. Scientists have devoted a large amount of work to studying the metabolic pathways of exogenous compounds in crops in recent years; however, the relationships between the agents in the detoxification system in xenobiotic metabolism are still not clear [12]. Interestingly, herbicide detoxification reactions of crops can be induced by herbicide safeners. Herbicide safeners are synthetic chemicals used to alleviate the phytotoxicity of herbicides to crops without reducing the efficiency of weed control [1, 13]. The use of herbicides in combination with safeners represents approximately 30% of the market share of global herbicides [14]. The importance of safeners in agricultural production has attracted considerable research on their modes of action. The difference in the herbicide metabolic rate between crops and weeds is essentially one of the mechanisms of herbicide selectivity. What is unique and interesting about safeners is that they can obviously simultaneously induce a series of key components of the detoxification pathway in plants, thus enhancing the metabolism, degradation ability and isolation of crops against herbicides and increasing the selectivity of weed control [1, 12, 15]. In the past several decades, the mechanisms by which safeners work have been the focus of attention for some scientists. However, until now, there have been no conclusions that have elucidated the modes of action of safeners, especially the molecular and genetic regulatory mechanisms and signaling pathways involved in safener action, which remain largely unclear.

The safener fenclorim (4, 6-dichloro-2-phenylpyrimidine, Fen) was developed to reduce the injury to rice (*Oryza sativa* L.) caused by chloroacetanilide herbicides. To ensure the safety of early stage rice, Fen is often formulated with pretilachlor [2-chloro-2′, 6′-diethyl-N-(2-propoxyethyl) acetanilide, Pre], which is one of the most widely used herbicides in rice fields in southeast Asian countries, but Pre represents phytotoxicity risk to rice [16]. Fen protects rice from injury by Pre mainly due to the acceleration of the metabolism of Pre [17]. Most studies have focused on the relationship between the induction of Fen and GST activity and the regulation of the expression of genes encoding GSTs [18–21]. Few studies have focused on detoxifying enzymes other than GSTs. As far as we know, only one study has investigated rice at the enzyme activity level, stating that Fen elevated the P450 content [22]. Moreover, many studies have been conducted on *Arabidopsis thaliana* [19, 23–26]. In a study of transcription levels, it has been shown that many genes encoding GSTs, UGTs and CYPs can be induced by Fen in both rice and *Arabidopsis thaliana* [12, 23]. However, it is believed that only a small number of upregulated genes have a strong capability to detoxify xenobiotics [12]; thus far, the exact genes related to Fen induction and the detoxification of Pre in rice have not been identified.

Additionally, both safeners and herbicides are biologically active xenobiotic compounds. Since herbicides can cause peroxidation in crops, there are still no clear results about the effects of safeners on ROS in rice. We now report a systematic study to illuminate the effects of Fen on the physiology of rice, the ability of rice to metabolize Pre, and the effect of Fen and Pre on rice gene expression at the transcriptome level. Here, we aim to (1) study the ability of Fen to enhance GST activity and the metabolic rate of Pre, (2) investigate the activities of antioxidant enzymes of rice seedlings on the Pre substrate in the presence of Fen and (3) perform global transcriptome studies in rice treated with Fen to identify the genes that are closely related to the mode of action of the Fen and Pre metabolic pathways.

## Results

### Effects of Pre and Fen on total chlorophyll

**Fig. 1:**
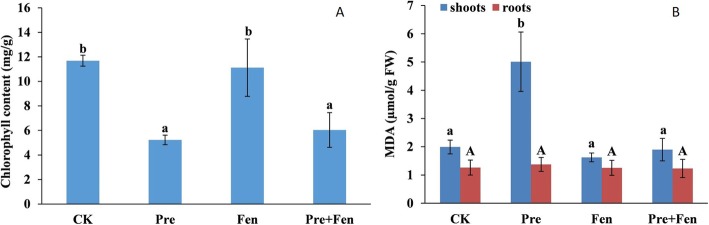
Effect of Pre and Fen on the total chlorophyll content and MDA. Three-day-old rice seedlings were used in control (CK), Pre (10 μM), Fen (10 μM) and Pre + Fen (10 μM + 10 μM) treatments and grown for 5 days. The bars represent the averages ± standard deviations of three replicates. Lowercase and capital letters indicate significant differences within each treatment in the shoots and roots of rice plants, respectively (*p* < 0.05), determined by LSD 0.05 test

The total chlorophyll content of rice plants was decreased by 55.6% compared to that of the control under exposure to Pre. Fen alone did not decrease the total chlorophyll content, and there was no difference between the treatments of Pre alone and Fen combined with Pre (Fig. 1A).

### Effects of Pre and/or Fen on oxidative stress and antioxidant enzymes

To evaluate the cellular damage caused by selected chemicals, the malondialdehyde (MDA) content in the leaf and root tissue was quantified. As shown in Fig. 1B, treatment with Pre alone caused a strong increase in the MDA contents of rice shoots, reaching an MDA content 2.5-fold that of the control. Interestingly, Fen did not increase the MDA content; in contrast, it reversed the accumulation of MDA caused by Pre when it was used in combination with Pre. However, neither Pre nor Fen stimulated MDA accumulation in the roots.

**Fig. 2:**
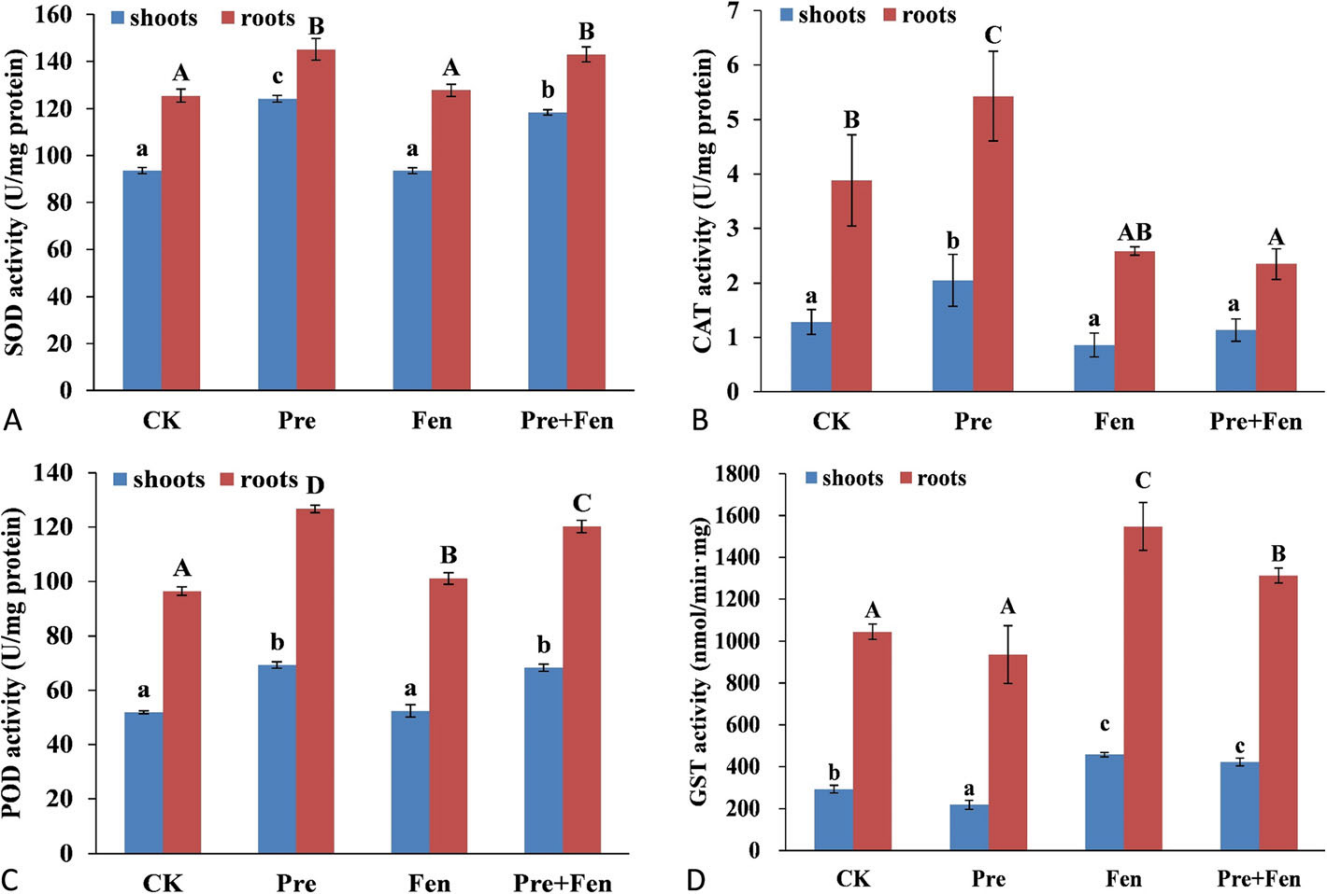
Effects of Pre and Fen on the activities of SOD, CAT, POD and GST. Three-day-old rice seedlings were used for control (CK), Pre (10 μM), Fen (10 μM) and Pre + Fen (10 μM + 10 μM) treatments and grown for 5 days. The bars represent the averages ± standard deviations of three replicates. Lowercase and capital letters indicate significant differences within each treatment in the shoots and roots of rice plants, respectively (*p* < 0.05), determined by LSD 0.05 test

The activities of SOD, CAT and POD in the rice shoots and roots were variable when treated with Pre and/or Fen. In general, the SOD, CAT and POD activities were intrinsically higher in the roots than in the shoots (Fig. 2A, B, C). The SOD, CAT and POD activities varied similarly in the rice shoots and roots under Pre and Fen exposure. Pre induced significant increases in the SOD, CAT and POD activities in both the shoots and roots. In the shoots, the SOD, CAT and POD activities increased by 32.7, 59.6, and 33.5%, respectively. In the roots, the SOD, CAT and POD values were increased by 15.6, 40.0 and 31.5%, respectively. Fen alone did not increase the SOD, CAT and POD activities in either tissue; in contrast, when Fen was used in combination with Pre, the SOD and CAT activities in the shoots were reduced by 4.9 and 80.5%, respectively, compared to the activities in shoots treated with Pre alone. The CAT and POD activities in the roots were reduced by 131.6 and 5.4%, respectively, compared to those in roots treated with Pre alone. However, the POD activity in the shoots and the SOD activity in the roots did not decrease significantly when Fen was combined with Pre compared with Pre alone.

### GST activity assay and effects of Pre and Fen

The intrinsic activity of GSTs in roots of rice was 3.6-fold that in the shoots of the control treatment. The Pre treatment significantly suppressed the activity of GSTs in the shoots, while the Fen treatment increased the activity by 56.5%, and the Fen combination with Pre increased the activity by 44.3%. Pre did not elevate GST activity in the roots compared to that of the control. Fen treatment significantly increased the activity of GSTs in the roots whether used alone or combined with Pre (Fig. 2D).

### Pre residue in rice shoots

**Fig. 3:**
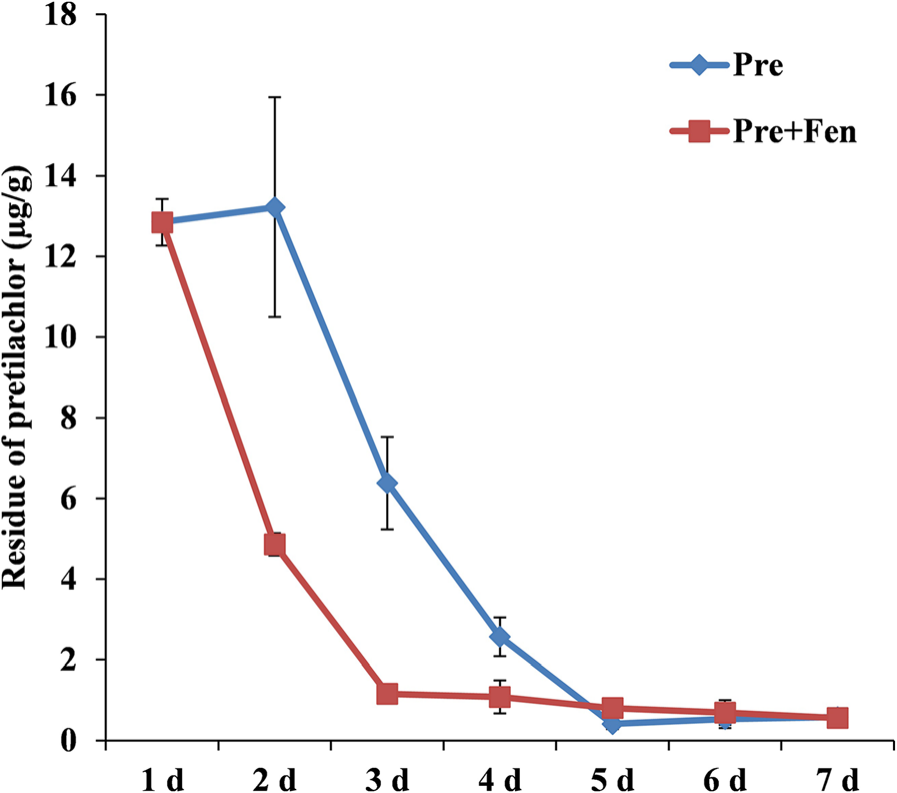
Residual dynamics of Pre in rice shoots. Six-day-old rice seedlings received the following treatments: Pre (10 μM) and Pre + Fen (10 μM + 10 μM). The data points are the averages ± standard deviations of three separate replicates

Fen did not affect the accumulation of Pre in the rice shoots in the first 24 h. Starting at 48 h, the Pre content showed a significant difference between the Pre and Pre + Fen treatments. When Pre was applied alone, the amount of Pre in the shoots was slightly increased at 48 h, after which it degraded from 72 h to 120 h. When the Pre and Fen mixture was applied, the Fen reduced the Pre content rapidly from 48 h to 120 h (Fig. 3).

### Illumina sequencing, assembly, and gene ontology

To investigate the gene expression profile involved in the Fen-mediated activation of the detoxification genes in rice, we compared the transcriptomes of 12 rice samples, including three biological replicates of the untreated control (CK) and Fen treatment for two time points (4 and 24 h).

Considering that Fen can elicit rapid xenobiotic responses but also monitor secondary events, the 4 h and 24 h transcripts were sequenced for 12 samples [12, 23]. After quality control and data cleanup, we obtained 84.37 Gb of clean data ranging from 20,920,063 to 29,230,636 reads per sample; 86.8% of the clean reads had a quality ≥30, and 98.0% of the clean reads were quality filtered and matched the Illumina quality requirements. The clean reads were then used to map the sequence of the rice genome sequence (Table 1). The proportion of clean reads in each sample mapped to the reference genome ranged from 71.8–76.3%. All raw-sequence read data were uploaded to the NCBI Sequence Read Archive (SRA, https://submit.ncbi.nlm.nih.gov/subs/sra/) with accession number PRJNA560668. A library was constructed after the sample was qualified. Finally, a total of 35,301 genes were obtained. Of these, 16,638 had lengths between 1000 and 300 nt, and 14,743 were longer than 1000 nt.

**Table 1:** Summary of rice transcriptome sequencing, assembly and comparison with reference genome

	Reference transcriptome
**Total clean reads**	284,404,035
**Clean bases**	84.37 Gb
**All reads (mapped genes)**	35,301
**Total mapped reads (% of reference genes)**	71.8–76.3%
**Multiple mapped reads (% of reference genes)**	2.94–4.88%
**Uniquely mapped reads (% of reference genes)**	67.81–72.82%

Gene Ontology (GO) analysis assigned 28,662 genes into 54 functional categories: 17 for cellular components (CCs), 17 for molecular functions (MFs), and 20 for biological processes (BPs) (Additional file 1: Figure S1). The most enriched subgroups in the CCs were “cell parts” (21,579 genes, 75.29%), followed by “cell” (21,555 genes, 75.20%) and “organelle” (19,691 genes, 68.70%). The most enriched subgroups in the MF category were “binding” (13,871 genes, 48.40%) and “catalytic activity” (12,970 genes, 45.25%). The most enriched subgroups in BP were “metabolic process” (15,693 genes, 54.75%), “cellular process” (13,512 genes, 47.14%) and “single-organism process” (11,396 genes, 39.76). Additionally, Kyoto Encyclopedia of Genes and Genomes (KEGG) analysis classified 7752 genes into 124 pathways.

### Functional analysis of the differentially expressed genes (DEGs)

The read counts of all genes are supplied in the Additional file 2: Table S1. The transcript levels were calculated by fragments per kilobase of transcript per million mapped reads (FPKM) (Additional file 3: Table S2). At 4 h, a total of 200 differentially expressed genes (DEGs) were found, among which 107 genes were upregulated and 93 were downregulated (Additional file 4: Table S3). At 24 h, a total of 81 DEGs were found, including 35 upregulated and 46 downregulated genes (Additional file 5: Table S4).

Based on the GO database, 168 DEGs (4 h) and 68 DEGs (24 h) were annotated. The DEGs at 4 h and 24 h were assigned to 54 functional subgroups, including 20 for “BP”, 17 for “CC”, and 17 for “MF” (Additional file 6: Figure S2). Among the DEGs, “catalytic activity” in the MF category and “metabolic process” in the BP category were significantly enriched in the Fen treatment relative to the CK.

Based on the known modes of action of herbicide safeners [1, 11, 27], we believe that the DEGs related to metabolism and signaling pathways were associated with herbicide metabolism, which can be strengthened by safeners. We selected the genes that were upregulated in the Fen treatment vs CK that had related functional annotations, including P450, GST, and UGT genes. The genes that were potentially involved in the herbicide detoxification process induced by Fen are listed in Table 2. There were 16 detoxification genes belonging to phase I and six belonging to phase II. In phase I, five genes coded for cytochrome P450s, four genes coded for oxygenases, and two genes coded for peroxidases. Some fenclorim-induced hydrolases (3 glycosyl hydrolases, 1 thioesterase, 1 lipase) may also be included in phase I. Two GSTs and five glycosyl transferases may contribute to phase II. Two GST genes belonging to the phi and tau classes were upregulated 24 h after Fen induction. Another important gene superfamily, the glycosyl transferases, whose members can catalyze reactions of toxicants with sugars to form hydrophilic compounds ready for further catabolism, included four members that were also induced by the Fen treatments at 4 h [28].

**Table 2:** Fenclorim-induced detoxification genes in rice

Phase/Gene ID	Fold	***P*** value	Description	Induced time
**Phase I**				
P450				
Os12g0150200	2.4	4.38E-03	Cytochrome P450, *CYP94C2b*	4 h
Os02g0703600	2.0	5.52E-05	Cytochrome P450, *C7IY83*	4 h
Os02g0204700	2.6	5.41E-04	Cytochrome P450, *CYP734A2*	24 h
Os06g0497275	2.1	6.74E-03	Cytochrome P450, *CYP71K14*	24 h
Os06g0497200	3.1	7.37E-03	Cytochrome P450, *CYP71D55*	24 h
Oxygenase				
Os03g0289850	1.4	2.09E-03	2OG-Fe (II) oxygenase superfamily	4 h
Os04g0497000	1.3	5.44E-05	Oxidoreductase activity	
Os04g0372700	1.2	6.51E-03	Putative quinone-oxidoreductase homolog	4 h
Os07g0561500	1.3	9.64E-03	Oxidoreductase activity	24 h
Peroxidase				
Os04g0688300	2.2	1.17E-03	Peroxidase	4 h
Os07g0676900	1.5	5.85E-04	Peroxidase	24 h
Glycosyl hydrolase				
Os10g0565200	2.5	7.92E-10	Glycosyl hydrolase family 14	4 h
Os04g0604300	2.1	4.69E-03	Glycosyl hydrolase family 16	4 h
Os11g0701100	3.1	5.43E-03	Glycosyl hydrolase family 18	4 h
Lipase/thioesterase				
Os01g0253900	1.1	5.31E-03	Lipase (class 3)	4 h
Os06g0143400	1.9	2.35E-05	Acyl-ACP thioesterase; Acyl-ATP thioesterase	24 h
**Phase II**				
GST				
Os10g0528651	1.3	5.10E-03	Glutathione S-transferase; *OsGSTU16*	24 h
Os01g0369800	1.7	5.42E-03	Glutathione S-transferase; *OsGSTF5*	24 h
Glycosyl transferase				
Os04g0206500	2.6	2.56E-06	UDP-glucoronosyl and UDP-glucosyl transferase	4 h
Os01g0880200	1.1	1.66E-03	Glycosyl transferase family 8	4 h
Os10g0555100	1.4	5.30E-04	Glycosyl transferase family 8	4 h
Os04g0556500	1.3	7.04E-03	UDP-glucoronosyl and UDP-glucosyl transferase	4 h
Phase IV				
Os04g0516600	1.7	3.33E-05	Beta-eliminating lyase; DegT/DnrJ/EryC1/StrS aminotransferase family	24 h
Os04g0516701	1.7	1.26E-04	Beta-eliminating lyase; DegT/DnrJ/EryC1/StrS aminotransferase family	24 h
Other				
Os04g0543900	2.0	5.78E-03	Glutamate/leucine/phenylalanine/valine dehydrogenase	4 h

### Quantitative real-time RT-PCR (qRT-PCR) validation of metabolizing enzyme genes

**Fig. 4:**
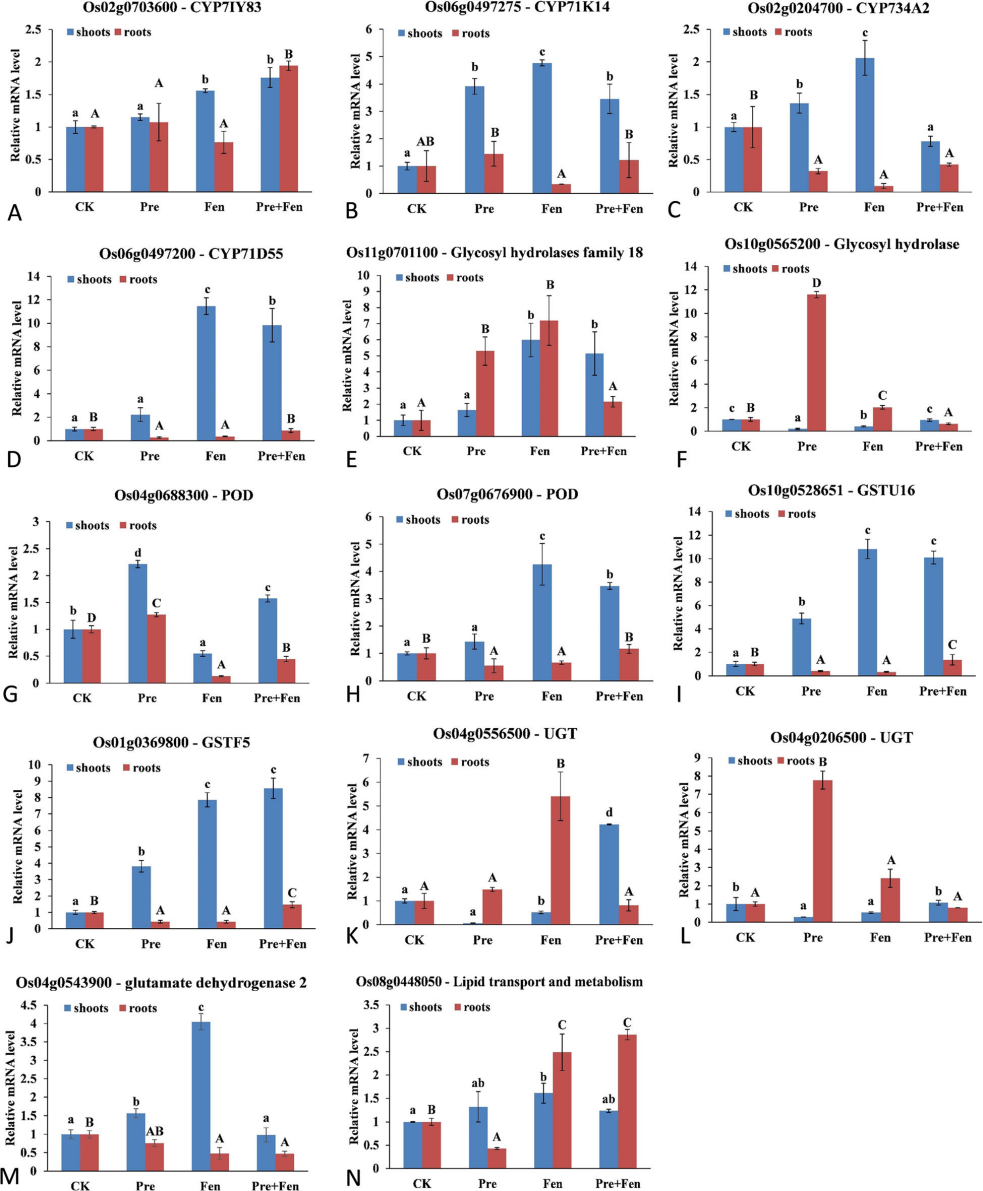
qRT-PCR validation for 14 upregulated genes induced by Fen and Pre in rice. The *actin* gene was used as the internal control. Lowercase and capital letters indicate significant differences within each treatment in the shoots and roots, respectively (*p* < 0.05), determined by LSD 0.05 test. The means and standard deviations from three biological replicates are shown

To identify and validate the genes induced by Fen, the expression of 14 upregulated candidate genes was validated by qRT-PCR analysis under Fen treatment, and the expression profiles of these genes as induced by Pre were also analyzed. Most of the selected genes had similar functional annotations, including P450 genes, GST genes, GT genes, POD genes, hydrolase genes, dehydrogenase genes and lipid transporter and metabolism genes (Fig. 4). The ten upregulated genes other than Os10g0565200, Os04g0688300, Os04g0556500 and Os04g0206500 showed higher expression levels in shoots exposed to Fen than in the control shoots, which was basically consistent with data from RNA-Seq, suggesting that most of the DEGs resulting from RNA-Seq were valuable for further analysis. These 10 upregulated genes included four P450 genes (*CYP71Y83*, *CYP71K14*, *CYP734A2*, and *CYP71D55*) (Fig. 4A, B, C, D), two GST genes (*GSTU16* and *GSTF5*) (Fig. 4I, J), one hydrolase gene (Fig. 4E), one POD gene (Fig. 4H), one dehydrogenase gene (Fig. 4M) and one lipid transporter and metabolism gene (Fig. 4N). Four of them, two hydrolase genes (Os11g0701100 and Os10g0565200) (Fig. 4E, F), one UGT gene (Os04g0556500) (Fig. 4K), and one lipid transporter and metabolism gene (Os08g0448050) (Fig. 4N), had significantly greater expression levels in the roots treated with Fen than in control roots. When plants were treated with Pre, seven genes (three P450 genes (Fig. 4B, C, D), two GST genes (Fig. 4I, J), one POD gene (Fig. 4G), and one dehydrogenase gene (Fig. 4M)) had significantly increased expression levels in the rice shoots, and five genes had significantly increased expression levels in the roots. Among them, the POD gene Os04g0688300 (Fig. 4G) was significantly upregulated by Pre in both the shoots and roots but was especially upregulated in shoots. Moreover, seven genes (three P450 genes (Fig. 4A, B, D), two GST genes (Fig. 4I, J), one hydrolase gene (Fig. 4E) and one POD gene (Fig. 4H)) that were upregulated when plants were exposed to Fen were expressed at even higher levels in shoots when plants were treated with Fen + Pre.

## Discussion

Herbicide safeners can alleviate the injury caused by herbicides to crops. Although there has been some progress in the description of the mechanism by which safeners work, their mode of action has not been fully clarified. Fen is a safener specifically developed for Pre, and the use of Fen could significantly increase the tolerance of rice to Pre. However, most relevant previous studies have focused on the detoxifying enzyme GST [17–19, 21, 26, 29, 30], and the other effects of Fen on rice plants remain elusive. The present study determined the total chlorophyll content, lipid peroxidation, and transcriptional expression induced by Fen.

Our study has shown that Pre causes a loss of chlorophyll content. The addition of Fen did not alleviate the chlorophyll content reduction, indicating that Pre disrupts the biosynthesis of chlorophyll and that the safener Fen does not affect the biosynthesis of chlorophyll. This finding may be one of the characteristics of this particular compound. This observation is also in line with the results that Fen alone does not affect rice growth. Nevertheless, when Fen was used in combination with Pre, the chlorophyll content did not recover, perhaps because the recovery of chlorophyll biosynthesis requires a much longer time period than was used in this work.

**Fig. 5:**
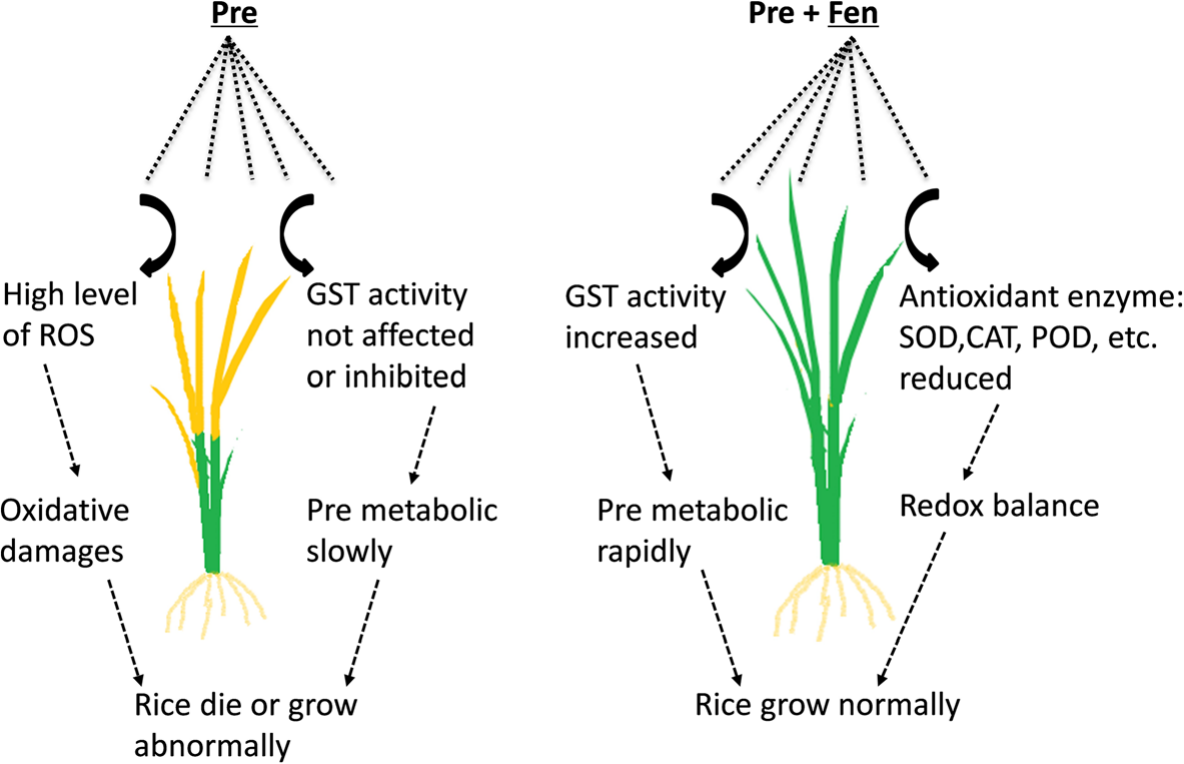
Schematic representation of the differential response in rice after treated with Pre or/and Fen

ROS have been linked to herbicide toxicity and cause the loss of membrane integrity via lipid peroxidation; lipid peroxidation is considered to be the most destructive molecular process in all organisms [31]. Antioxidant systems play a key role in maintaining a balance between ROS production and scavenging in plants. The antioxidant response in crops to herbicides has been a recent subject of investigation, and it has been established that most herbicides can induce the formation of ROS, but there are very few studies about the effects of safeners on crop antioxidant systems. Therefore, another aim of our study was to clearly define the effect of Fen on lipid peroxidation and antioxidant systems in rice. In the present study, we determined the MDA content and activities of SOD, CAT and POD, which are indicators of lipid peroxidation and oxidative stress in plant cells, respectively. Our study showed that Pre can cause high levels of ROS. The antioxidant activities of the enzymes SOD, CAT and POD were significantly increased by Pre, and Fen alone did not increase the SOD, CAT and POD activities in either the shoots or the roots. In contrast, Fen ameliorated the oxidative stress induced by Pre. In addition, Fen also reduced the accumulation of MDA caused by Pre to normal levels in rice shoots, consistent with the results of antioxidant defense. Since Pre can cause serious harm to rice seedlings, it is not surprising that the rice seedlings suffered from severe oxidative stress caused by Pre, but the effect of Fen on the antioxidant systems in rice suggested that the defensive antioxidative properties are also one manner in which Fen protects rice from injury caused by Pre. In a previous study related to the effects of chloroacetamides and safeners (mefenpyr and dichlormid) on human blood cells, the safeners alone did not elicit any changes in oxidative stress but instead reduced the lipid peroxidation induced by the chloroacetamide herbicides [32]. This result, together with ours, suggests a role of safeners as antioxidants both in human blood cells and in rice (Fig. 5).

Based on previous studies, it can be concluded that enhancing the rate of metabolism and/or the sequestration of herbicides are the major mechanisms by which safeners protect plants from injury [1, 33]. With regard to the effects of Fen on the accumulation and persistence of Pre residues in rice shoots, we found that Fen did not interfere with the uptake of Pre but induced a rapid reduction in Pre persistence. These results were expected since it has come to light that Fen is able to protect rice from Pre injury by enhancing its metabolism [34, 35]. Furthermore, the detoxification of Pre in rice is highly correlated with GST activity. Deng et al. [18] and Wu et al. [21] observed that Fen significantly increased GST activity in rice. The results of this study also found that Fen could significantly induce the activity of GST in both shoots and roots, which is consistent with the results of previous studies. Our results showed that Fen can strengthen the metabolism of rice by increasing the activity of detoxification enzymes, such as GST (Fig. 5).

The molecular mechanisms of safener action may involve complex interactions between multiple signals and detoxification pathways, which can then protect plants from herbicides and other exogenous compounds [1]. Several studies have investigated the mechanism of safeners in model plants and very few graminaceous crops at the transcriptome level, but the exact mechanism remains unclear. We used RNA-Seq to identify new candidate genes involved in the Fen response in rice. In view of the time dependence of mRNA expression, we set two time points for the RNA-Seq approach. In total, 107 and 35 genes were upregulated at 4 h and 24 h, respectively. Among the 142 differentially upregulated genes, 26 potentially encoded detoxification enzymes related to herbicide metabolism, and most of these 26 were upregulated at 24 h. This result is consistent with the results of previous studies showing that safeners induce a series of components of the detoxification system in *Arabidopsis thaliana* [11, 23]. Similar results were also reported in rice and sorghum. Most of the genes that encode detoxification enzymes, including P450s, GSTs, and UGTs, were upregulated by fluxofenim in sorghum [33]. Brazier-Hicks et al. treated rice seedlings with Fen and found that many enzymes, such as CYP, GST and UGT, had upregulated expression [12]. In the current study, 5 CYP genes, 2 GST genes, 4 GT genes and several other detoxification genes were upregulated when the rice was treated with Fen. Among them, only one gene, *CYP734A2,* was previously found to be induced by Fen [12]. We did not find other overlap in the inducible genes that were identified between Brazier-Hicks’s study and our results, and far fewer inducible genes were identified in this study than in a previous report [12], which may be due to the differences in species and the decreased dosage of Fen (10 μM vs 100 μM). Since it is inferred that only a small number of safener-induced enzymes possess high detoxifying activities towards xenobiotics [12], the small number of genes induced by Fen in this study is not a bad thing. Combined with the following qRT-PCR validation, we identified at least four P450 genes, two GST genes and one UGT gene that appeared to be related to the Fen mode of action, some of which were also induced by Pre and Pre + Fen. In particular, the two GST genes (*GSTU16* and *GSTF5*) exhibited strong inducible expression. This result indicated that these genes may be involved in the metabolism of Pre, but Fen itself can also be metabolized by GSTs [25]. The function of these enzymes in metabolizing Pre needs further study.

## Conclusion

This study further confirmed that Fen can protect rice from the damage caused by Pre by enhancing the metabolism of Pre in rice. Moreover, we did not find any oxidative damage in rice caused by Fen; rather, Fen reduced lipid peroxidation and the ROS production induced by Pre. These findings provide new insight into the antioxidant activity of Fen, and we suppose that antioxidative-defensive properties are another mode of action of Fen. Transcriptome analysis indicated that Fen induced many response genes related to the detoxification pathway, including P450, GST, and GT. qRT-PCR results showed that some of them were concurrently induced by Fen and Pre. We believe that there is great value in the further study of these DEGs. These results should be useful for elucidating the mode of action of Fen.

## Methods

### Plant material and growth conditions

Rice seeds of variety 9311 (acquired from the China National Hybrid Rice R&D Center and identified by associate research fellow Li Li) were sterilized in 3% sodium hypochlorite for 5 min, washed in distilled water three to four times, soaked in tap water for 24 h, and then germinated at 28 °C in the dark for 48 h [36]. The germinating seeds were transferred into a plastic case containing Hoagland nutrient solution [37] under a 14 h/10 h light/dark cycle at 28/25 ± 1 °C (day/night).

For studies of the residue dynamics of herbicides in rice, the rice plants were divided into four treatments 6 days later: one treatment was left as the control, the second treatment was 10 μM Pre, the third treatment was 10 μM Fen, and the fourth treatment was Pre + Fen (10 μM + 10 μM). The design of compound dosage was from the literature [23]. For chlorophyll, MDA and antioxidant activity analyses, rice seedlings were treated with herbicide and/or safener 3 days after the rice seeds were inoculated and cultured continually for 5 days. For transcriptome analysis and qRT-PCR determination, rice seedlings were cultivated to the 3 leaf stage and were then treated the same as in the residue dynamics experiment. Rice shoots and roots were harvested after 4 and 24 h to extract the total RNA for the transcriptome analysis and qRT-PCR determination [23]. A total of 12 samples for transcriptome analysis, including three biological replicates of the untreated control (CK) and Fen treatment for two time points (4 and 24 h), were collected and frozen in liquid nitrogen and stored at − 80 °C until RNA extraction.

All the chemicals, including Pre and Fen, that were involved in these experiments were dissolved in acetone and diluted with water containing 0.1% Tween-80. The control treatments contained the same amount of acetone and Tween-80. The shoots and roots of the rice seedlings were collected separately and stored at − 70 °C before analysis.

### Analysis of chlorophyll and lipid peroxidation

Chlorophyll extraction and content assays were performed according to the protocols described by Porra et al. [38]. Fresh rice flag leaves (0.1 g) were weighed and cut into 1 mm filaments, added to 10 cm^3^ of 80% acetone and stored at 4 °C for 72 h. The absorbance of the solution was then determined at 663.6 nm and 646.6 nm using an ultraviolet spectrophotometer (UVmini-1240, Shimadzu, Japan), and the total chlorophyll content was calculated.

Lipid peroxidation was determined in terms of MDA content according to the method described by Wang et al. [39]. Fresh tissue (0.5 g) was weighed and ground in liquid nitrogen and then extracted in 3 cm^3^ of 0.67% trichloroacetic acid (TCA) solution. The homogenate was centrifuged at 10,000 g for 30 min, and then 2 cm^3^ of the supernatant was mixed with 2 cm^3^ of 0.5% TCA (the solvent was 20% TCA). The mixture was placed in boiling water for 30 min, cooled to room temperature and centrifuged at 15,000 g for 5 min. The absorbance of the supernatant was measured at 532 nm, and the nonspecific absorbance value at 600 nm was subtracted (0.5% thiobarbital acid (20% TCA in solvent) was blank).

### GST and antioxidant activity analysis

Extraction of GST and GST activity assays were performed according to the protocols described by Li et al. [40]. The activities of CAT, POD, SOD were determined with assay kits (Jiancheng, Nanjing, China) according to the manufacturer’s instructions. The protein content was determined by the Bradford method [41].

### Determination of Pre in rice

Extraction of Pre from rice was performed using a method described previously [35]. Shoot samples (2 g) were powdered in liquid nitrogen and extracted and homogenized with 20 cm^3^ of methanol and water (4:1, V/V) for 3 min. The mixture was then centrifuged at 5000 g for 15 min, and the supernatant was extracted twice with 30 cm^3^ of CHCl_3_. The organic phase was evaporated to dryness, and the residual was rinsed with 1 cm^3^ of water/acetonitrile (3:7 v/v). The samples were qualitatively analyzed using an HPLC equipped with a C_18_ reversed-phase column. Pre was quantified by a UV detector at a wavelength of 210 nm with a mobile phase (water: acetonitrile, 3:7 v/v) at a flow rate of 1 cm^3^/min.

### Library preparation for transcriptome sequencing and bioinformatics analysis

A total of 12 samples were collected (three biological replicates × two treatments × two time points). Total RNA was extracted from each sample using RNAiso Plus (TaKaRa Biotech, Dalian, China) following the manufacturer’s recommendations. The quality and quantity of the total RNA were analyzed using an Agilent Bioanalyzer 2100 system (Agilent Technologies, Santa Clara, CA, USA).

cDNA library construction, including mRNA isolation, mRNA fragmentation, cDNA synthesis, and PCR amplification, was performed at Beijing BioMarker Technologies (Beijing, China). Sequencing libraries were generated using the NEBNext, Ultra RNA Library Prep Kit for Illumina (Illumina, Inc., San Diego, CA, USA) according to standard methods. In brief, mRNA was enriched from total RNA with magnetic beads carrying poly-T oligo-nucleotides, and then randomly broken into short sequences in fragmentation buffer. A random hexamer primer and M-MuLV Reverse Transcriptase (RNase H free) were used for synthesis of the first-strand of cDNA. Subsequently, the cDNA was amplified by PCR with DNA polymerase I after the treatment with RNase H. Fragments (200–250 bp) of the PCR product were selected using AMPure XP system (Beckman Coulter, Beverly, USA). Purified double-strand cDNA was then used for end repair, adding an A-tail and connecting the sequencing beads. AMPure XP beads were used to select the bead size. PCR products were purified (AMPure XP system), and the quality of the cDNA library was evaluated by the Agilent Bioanalyzer 2100 system. Finally, paired-end reads were sequenced on an Illumina HiSeq 2500 platform, and 150 bp paired-end reads were obtained.

The raw data were filtered to remove adaptor sequences and low-quality reads, and high-quality clean data were subsequently obtained. The Q20, Q30, GC content and sequence duplication level of the clean data were calculated. The mapped data were obtained as a sequence alignment between the clean data and the specified reference genome (ftp://ftp.ensemblgenomes.org/pub/plants/release-24/fasta/oryza_sativa/) using Tophat2 software [42].

### DEG analysis

The read count for each gene was obtained from the mapping results, and the quantification of the gene transcript abundance was measured by FPKM [43]. Gene expression differences between the control and the Fen-treated plants were analyzed using the DESeq R package. *P* value calculations and false discovery control were performed using Benjamini’s approach [44]. Genes with an adjusted *P* value < 0.05 and |log2 (fold change) | ≥ 1 were considered differentially expressed. The DEGs were further subjected to GO enrichment and KEGG pathway enrichment analyses. GO (http://geneontology.org/) analysis was implemented by a hypergeometric test [45]. KEGG (http://www.genome.jp/kegg) enrichment analysis was performed by comparing the genes of metabolic pathways or signal transduction pathways with the whole genomic background. GO or KEGG terms with q < 0.05 were considered significantly enriched.

### Transcript analysis by qRT-PCR

To verify the expression of the DEGs, 14 candidate detoxification genes were selected and their transcription was quantified by qRT-PCR. Total RNA was extracted from samples using TRIzol. cDNA was synthesized using a TransScript All-in-One First-Strand cDNA Synthesis SuperMix for qPCR (TransGen Biotech, Beijing, China) following the instructions. The qRT-PCR mix (20 mm^3^) contained 10 mm^3^ of 2 × TransStart Tip Green qPCR SuperMix (TransGen Biotech, Beijing, China), 0.4 mm^3^ of each primer (10 μM), 0.4 mm^3^ of Passive Reference Dye II, 2 mm^3^ of cDNA, and 6.8 mm^3^ of nuclease-free water.

Primers were designed using the online software Primer3, and the sequences of the primers are listed in Additional file 7: Table S5. *Actin* was used as a reference gene. qRT-PCR was performed by an ABI 7300 plus real-time PCR system (Applied Biosystems, Foster City, CA, USA). The qRT-PCR program consisted of 94 °C for 30 s, 40 cycles of 94 °C for 5 s and 60 °C for 34 s and, finally, 60 °C for 15 s. The expression of each gene relative to that of the control was calculated in accordance with the 2^-ΔΔCt^ method [46]. Each experiment consisted of three biological replicates.

### Statistical analysis

Three biological replications were carried out independently in this study. The data are expressed as the means ± standard deviations of three replicates. The significance of the differences between the treatments was statistically analyzed by one-way analysis of variance (ANOVA) based on the least significant difference (LSD) at the 5% level using SPSS software 20.0.

## Supplementary information

**Additional file 1: Figure S1.** GO classification and statistical results for all genes. The genes were summarized in biological process, cellular component and molecular function terms. A total of 28,662 genes were categorized.

**Additional file 2: Table S1.** Read counts of all genes.

**Additional file 3: Table S2.** Read FPKM per gene.

**Additional file 4: Table S3.** List of DEGs between Fen treatment and CK at 4 h.

**Additional file 5: Table S4.** List of DEGs between Fen treatment and CK at 24 h.

**Additional file 6: Figure S2.** GO classification and statistical results for DEGs at 4 h (A) and 24 h (B) of treatment. The genes were summarized in biological process, cellular component and molecular function terms. A total of 168 differentially expressed genes at 4 h of treatment and 68 differentially expressed genes at 24 h of treatment were annotated.

**Additional file 7: Table S5.** Primer pairs used for qRT-PCR verification of gene expression in rice.

## Data Availability

The sequencing data generated in current study were deposited in the NCBI Sequence Read Archive (SRA) database (SRA, https://submit.ncbi.nlm.nih.gov/subs/sra/) under the accession number PRJNA560668. The datasets generated or analyzed during this study are included in this published article and its supplementary information files.

## Abbreviations

ABC: ATP-binding cassette
ANOVA: One-way analysis of variance
APX: Ascorbate peroxidase
BP: Biological process
CAT: Catalase
CC: Cellular component
CYP: Cytochrome P450 monooxygenase
DEGs: Differentially expressed genes
Fen: Fenclorim
FPKM: Fragments per kilobase of transcript per million mapped reads
GO: Gene Ontology
GSTs: Glutathione-S-transferase
GTs: Glycosyltransferase
KEGG: Kyoto Encyclopedia of Genes and Genomes
KOG/COG: Clusters of Orthologous Groups of proteins
MDA: Malondialdehyde
MF: Molecular function
POD: Peroxidase
Pre: Pretilachlor
qRT-PCR: Quantitative real-time PCR
RNA-Seq: RNA sequencing
ROS: Reactive oxygen species
SOD: Superoxide dismutase
TCA: Trichloroacetic acid
