# Supplementary figures and images for: Effects of fenclorim on rice physiology, gene transcription and pretilachlor detoxification ability
Source: BMC Plant Biol. 2020 Mar 6;20:100. doi: 10.1186/s12870-020-2304-y (PMC7059400; doi:10.1186/s12870-020-2304-y)

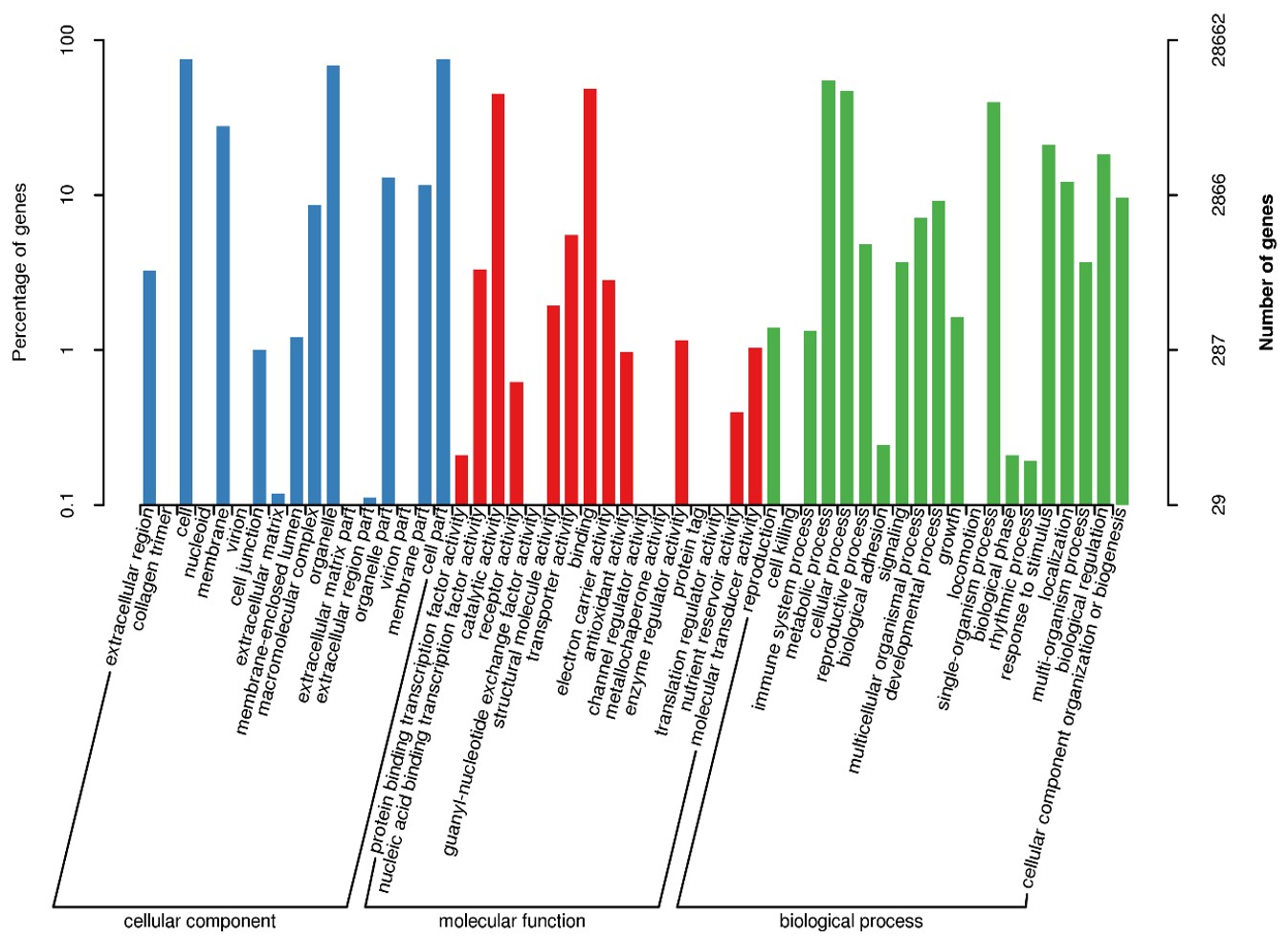

Supplement: Supplementary file 1 — Additional file 1: Figure S1. GO classification and statistical results for all genes. The genes were summarized in biological process, cellular component and molecular function terms. A total of 28,662 genes were categorized. [file 12870_2020_2304_MOESM1_ESM.jpg]

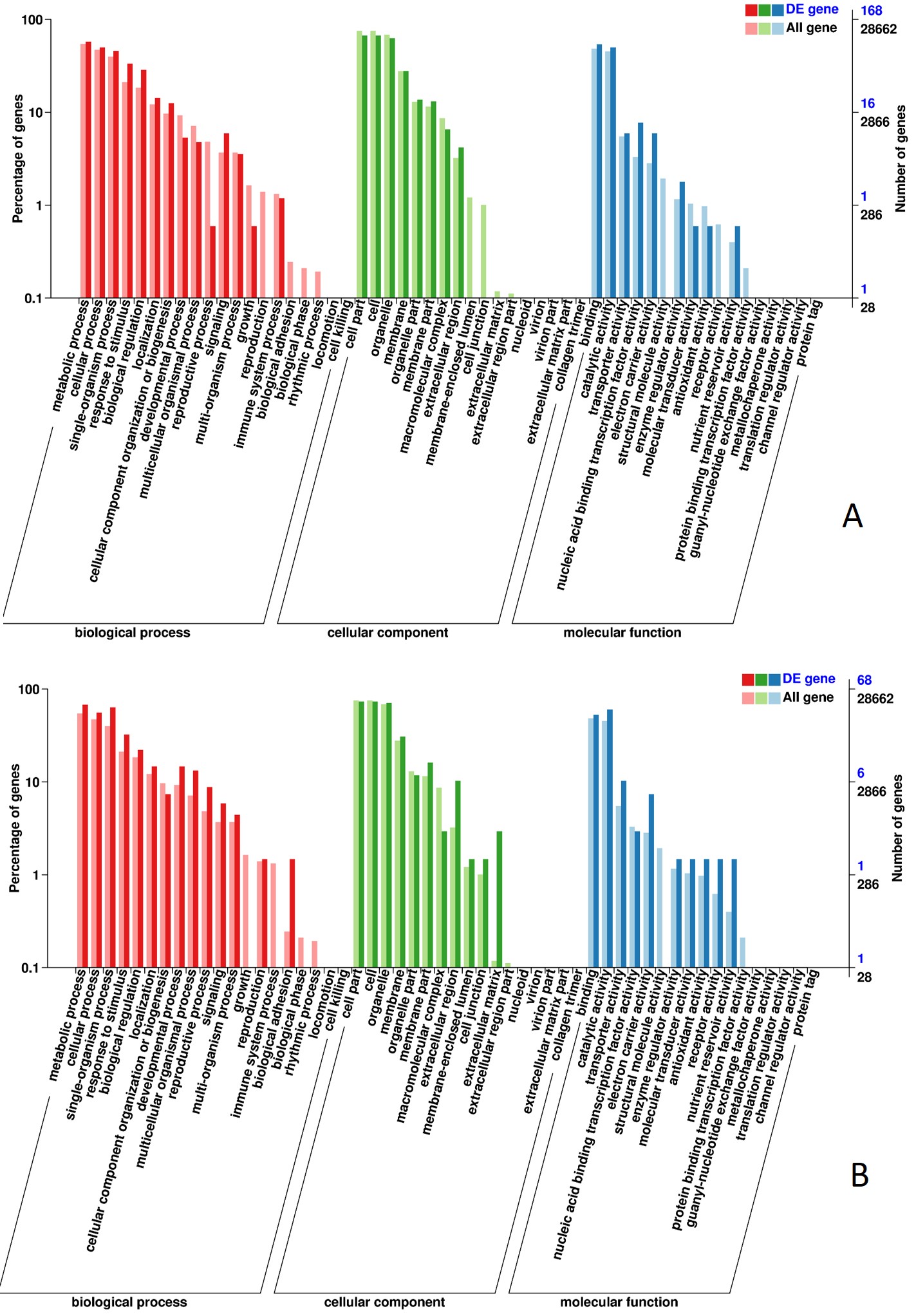

Supplement: Supplementary file 6 — Additional file 6: Figure S2. GO classification and statistical results for DEGs at 4 h (A) and 24 h (B) of treatment. The genes were summarized in biological process, cellular component and molecular function terms. A total of 168 differentially expressed genes at 4 h of treatment and 68 differentially expressed genes at 24 h of treatment were annotated. [file 12870_2020_2304_MOESM6_ESM.jpg]
