# Supplementary material for: Effects of fenclorim on rice physiology, gene transcription and pretilachlor detoxification ability
Source: BMC Plant Biol. 2020 Mar 6;20:100. doi: 10.1186/s12870-020-2304-y (PMC7059400; doi:10.1186/s12870-020-2304-y)
Supplement: Supplementary file 7 — Additional file 7: Table S5. Primer pairs used for qRT-PCR verification of gene expression in rice. [file 12870_2020_2304_MOESM7_ESM.docx]

| Gene ID | Primers | Sequence (5’ to 3’) |
| --- | --- | --- |
| Os02g0703600 | F | CTGAAGGTGAAGGAGAGGATG |
|  | R | AGATTGCCAAGGAGAAAGAGG |
| Os06g0497275 | F | CCTAGCAGTGAGGCCCATAG |
|  | R | GGCAACCACTTCGAGTTCAT |
| Os02g0204700 | F | ATTGATGGTGTGGGTGTGTG |
|  | R | GTTGAGGTTGTCCGGGTAGA |
| Os06g0497200 | F | TACTGCCTCCTCACGCTCTT |
|  | R | GATCTCTCTAGCCGCAATGG |
| Os11g0701100 | F | CATCCATGTGAGGATGTTCG |
|  | R | CGAACTGCAGCATCTCGTAG |
| Os10g0565200 | F | CGGCGACTCCTGCAAGTAAG |
|  | R | CTACGGTAAACATTGCCGGTC |
| Os04g0688300 | F | GCTTCGCTGCCTCTGCTTAA |
|  | R | AAGGCCATACTGGCGAGACC |
| Os07g0676900 | F | TACACCAACCTGCTGTCGAA |
|  | R | AGCAGACGAGCCTGATTTGT |
| Os10g0528651 | F | CCTTGGAGCACTCCTTGAAC |
|  | R | AATCGACGAATTGCCTCAAC |
| Os01g0369800 | F | CGGCCCTCGAACTTGTTGAT |
|  | R | CTGGAGGAGTCGGCAATGGT |
| Os04g0556500 | F | GCAGAGCTGGTGTGCAAATA |
|  | R | CGAAGTCATCCAAGCCTTTC |
| Os04g0206500 | F | GGTTGCGATGCCTCAGTCAG |
|  | R | TCCACTGCCCTCTTCCCATC |
| Os04g0543900 | F | TCTCTGTTCAGGTGGAATGC |
|  | R | TCAAACCACAATCCTCACCTC |
| Os08g0448050 | F | TGTCGAACCGCTTCATGAC |
|  | R | CATGTTCCACGTCTACTCCC |
